# Amyloid-beta fibrils as active contributors to synaptic dysfunction in Alzheimer’s disease

**DOI:** 10.1038/s41401-026-01808-7

**Published:** 2026-06-12

**Authors:** Nunzia Maisto, Giorgia Ciufolini, Sepideh Dashtiani, Lorenzo Casoli, Almerinda Di Venere, Marilena Carbone, Dalila Mango

**Affiliations:** 1 School of Pharmacy, Department of Biology, University of Rome ‘Tor Vergata’, 00133 Rome, Italy; 2 Department of Chemical Science and Technologies, University of Rome ‘Tor Vergata’, 00133 Rome, Italy; 3 Department of Physiology and Pharmacology “V. Erspamer”, Sapienza University of Rome, 00185 Rome, Italy; 4 Department of Experimental Medicine, University of Rome ‘Tor Vergata’, 00133 Rome, Italy

**Keywords:** Alzheimer’s disease, Aβ fibrils, lipid-protein interaction, neurotoxicity, synaptic plasticity

## Abstract

Alzheimer’s disease (AD) is a neurodegenerative disorder that is characterized by the accumulation of amyloid-beta (Aβ) aggregates, in the form of fibrils and plaques. While it has largely been stated that Aβ oligomers are the main toxic species, significant evidence indicates that fibrils may also be relevant to AD pathogenesis. Notably, evidence indicates that while fibrils, through direct interaction with neuronal membranes, contribute to synaptic dysfunction and cellular damage, no direct evidence between fibrils and their impact on neuronal functions, including plasticity, was investigated. This study inquired into the impact of Aβ fibrils complex on neuronal function and membrane integrity, shedding light on their contribution to synaptic defects. Aβ fibrils were generated from Aβ_1-42_ oligomers, and their effects were evaluated on synaptic plasticity in ex vivo hippocampal slices from both female and male mice. Compared to Aβ_1-42_ oligomers, fibrils induced more severe damage in synaptic plasticity, emphasizing their potent neurotoxicity effect underlying cognitive decline. Additionally, using a liposomal model to examine fibril-membrane interactions, it was observed that Aβ fibrils are able to affect membrane fluidity compared to the Aβ_1-42_ oligomeric species, indicating that the size and aggregation state of Aβ fibrils are crucial for their toxicity. These findings challenge the view that oligomers are the primary toxic species in AD, showing that Aβ fibrils also play an active role in cellular dysfunction, promoting a synaptic dysfunction correlated to the cognitive impairment observed in AD.

## Introduction

Alzheimer’s disease (AD) is a slowly progressive neurodegenerative disorder that is characterized by the presence of amyloid-beta (Aβ) plaques and neurofibrillary tangles in the brain. According to the amyloid cascade hypothesis, Aβ accumulation in the form of curvilinear and senile plaques, composed of Aβ fibrils, is fundamentally involved in the pathogenesis of AD [1–3]. However, failed clinical trials, aimed exclusively at reducing plaque burden, have refocused the research on soluble Aβ oligomers, which are globally accepted as major contributors to the early synaptic dysfunction associated with AD [1].

Despite this emphasis on oligomers, Aβ fibrils constitute a key pathological aspect of AD, and knowledge about the presentation of Aβ fibrils and their interactions with cellular components, particularly the neuronal membrane, is paramount to understanding the pathogenesis of AD [4]. Structurally, Aβ fibrils are primarily made up of, and represented as a highly ordered β-sheet structure, which then assembles into straight and unbranching fibrils. Once formed, they have the potential to physically interact with neuronal membranes and alter membrane integrity, as well as initiate calcium dysregulation within synaptic function [5, 6]. Additionally, Aβ fibrils are structurally polymorphic and contain multiple “strains” with different toxicities, seeding potential and affinities with biological membranes [7]. The heterogeneity of the fibrils may also help to explain the varying rates of progression and differences in regional vulnerability present in early AD, and implicate fibrils’ contributory relationship to the advent of the AD pathology [7].

The relevance of Aβ oligomers in the ethology of AD has been revealed, while the role of amyloid fibrils remains elusive. Several studies have extensively investigated the functional role of Aβ oligomers, revealing a concentration-dependent effect at the synapse [8, 9]. For instance, the physiological presence of Aβ at picomolar concentration has a central function in the physiological synaptic plasticity induction as well as in the modulation of neurotransmission and protection from external aggressive substances [10, 11]. Contrariwise, increased concentration, in the order of nanomolar, impairs the synaptic plasticity function, affecting neurotransmission and AMPA and NMDA receptors’ synaptic expression and leading to synaptic plasticity impairment in the hippocampus [12–17], a brain area primarily affected in AD.

Together, these findings demonstrate that Aβ oligomers exert a dual concentration-dependent effect, enhancing our understanding of their functional role in AD-related synaptic dysfunction. On the other hand, in the AD brain, different Aβ forms, including monomers, oligomers, protofibrils, and fibrils coexist, collectively impacting synapses and cells’ homeostasis [7, 18–20].

Fibrils, traditionally viewed as inert end-products, actually represent the final stage in a dynamic aggregation cascade that begins with monomer misfolding and evolves into higher-order assemblies and mature plaques [21, 22]. Recently, the therapeutic authorization of Lecanemab, a humanized monoclonal antibody specifically designed to bind to soluble Aβ protofibrils, further validates the therapeutic relevance of intervening in the early and intermediate stages of Aβ aggregation [23]. Indeed, inhibiting fibril formation and maturation may reduce amyloid load and damage, preventing neuronal death. Nonetheless, the complex mechanism whereby the different amyloid forms, including the fibrils, induce functional alterations in the brain is undisclosed.

In this study, using a multidisciplinary approach including electrophysiology, Scanning Electron Microscopy (SEM), and fluorescence spectroscopy, we explore the effects on synaptic function of a different heterogeneous complex of Aβ fibrils, obtained from fibrilization of Aβ_1-42_ at different aggregation time points. Specifically, by electrophysiological recordings, we have assessed the impact of Aβ fibrils on synaptic plasticity in an ex vivo hippocampal model [13, 14], while by SEM we have evaluated the Aβ fibrils’ structure distribution in our experimental condition and Aβ fibrils’ interaction with lipid membranes, particularly using a liposome-based model.

## Materials and methods

### Animals

All animals were handled in compliance with the national (D.Lgs 26/2014) and international guidelines for animal welfare (European Communities Council Directive 2010/63/EU). All efforts were approved by the appropriate institutional and state authorities of EBRI Rita Levi-Montalcini Foundation. In compliance with the Italian law and EU directives, all efforts were made to minimize the number of animals used and suffering according to the principle of 3Rs. Animal studies are reported in compliance with the ARRIVE guidelines [24]. C57/Bl6J mice were housed at the EBRI Rita Levi-Montalcini Foundation (Rome, Italy).

### Drugs and chemicals

Amyloid β_1–42_ peptide (Aβ_1-42_) was purchased from GenScript (USA). Dimethyl sulfoxide (DMSO; CAS No. 67-68-5), laurdan, ThT, and phosphate buffer saline (PBS, 1 tablet/200 mL) were purchased from Sigma Aldrich-Merck KGaA (USA). 1-Palmitoyl-2-oleoyl-sn-glycero-3-phosphocholine (POPC), cholesterol, phosphatidylserine, and ovine ganglioside GM1, were obtained from Avanti Polar Lipids (Birmingham, AL, USA).

### Aβ fibril preparation

Aβ_1–42_ working solutions were prepared by diluting the initial peptide batch 1 mg with DMSO (according to the datasheet, to obtain the oligomer solution). The obtained 500 µM Aβ_1–42_ stock was diluted with PBS to obtain a final 4:1 PBS:DMSO ratio. Specifically, 30 µL of stock solution was mixed with 120 µL of PBS, yielding a final peptide concentration of 100 µM. The samples were incubated at 37 °C for 61 h, and aliquots were also collected at defined fibrillization time points (t0, t20, t24, t38, t46, t61), where t0 represents the time point collected immediately after dilution and incubation.

### Evaluation of Aβ fibrils preparation by ThT assay

Fibrils formation evolution versus time of incubation at 37 °C was monitored through the analysis of ThT fluorescence. In particular, a stock solution of 60 μM ThT in PBS was prepared and stored at 4 °C. Working solution was prepared by diluting 5 times in PBS the ThT stock, thus reaching a final ThT concentration of 5 μM. In the assay the protein sample was added to the working solution in the amount necessary to reach a 5 μM final protein concentration so as to have [Aβ_1-42_]:[ThT] = 1:1. ThT fluorescence was monitored with a PC1 spectrofluorimeter (ISS Inc, Urbana-Champaign, IL USA) exciting the samples at *λ* = 440 nm and collecting the emission spectra in the range 460–600 nm.

### Slice preparation and incubation with Aβ fibrils

We prepared thin slices (350 μm) of the hippocampus from C57Bl6/J mice, following the method described by Cho and colleagues [25]. After resting for an hour in artificial cerebrospinal fluid (aCSF) enriched with oxygen (95%) and a small amount of carbon dioxide (5%), the slices were placed in a Petri dish for 30 min. During this time, they were bathed either in aCSF containing Aβ_1–42_ oligomers (final dilution 1:1000) [13, 14] or in solutions containing fibrils at the same dilution (1:1000), that had been allowed to form for different time points (t0 to t61). Following the incubation, extracellular field recordings were performed to assess synaptic plasticity.

### Electrophysiology

Extracellular field recordings were performed to assess synaptic plasticity using a stimulating electrode in the stratum radiatum to stimulate the Schaffer collateral pathway of the hippocampus (CA1–CA3) for inducing long-term potentiation (LTP). After placing the electrode in the Schaffer collateral pathway, the stimulus intensity was first adjusted to induce a field excitatory postsynaptic potential (fEPSPs) at about half the maximal fEPSP amplitude. A 20-min baseline recording was performed, then LTP was induced by the Theta Burst Stimulation (TBS) procedure that involves 4 very short bursts, at a frequency of 100 Hz, that contained an interburst interval of 200 ms or 0.2 s. After the TBS stimulation, fEPSPs were recorded for a further 1 h, and for the analysis the fEPSP amplitude was normalized to baseline. An additional aspect of the experimental paradigm was to measure paired-pulse ratio (PPR) by presenting a pair of stimuli at various time intervals (50, 100, 200, 300, 400, 500 ms) and calculating it as the amplitude of the second fEPSP over the amplitude of the first fEPSP.

### Scanning electron microscopy (SEM) imaging

The morphological characterization of Aβ_1-42_ after 61 h of fibrillation was performed using Scanning Electron Microscopy (SEM). To prepare samples, few droplets of the solution were deposited onto a clean silicon wafer prepared for SEM analysis. After the Aβ had dried, any remaining PBS was removed by rinsing the wafer with four drops of deionized water (2 µL each), followed by gentle drying using a nitrogen (N_2_) stream. This washing procedure was repeated three times. Silicon wafers were precleaned by sonication in ethanol followed by drying with a stream of nitrogen.

Imaging was performed using a SUPRA™ 35 Field Emission Scanning Electron Microscope (Carl Zeiss SMT, Oberkochen, Germany) at an acceleration voltage ranging from 1.5 to 7 kV, using imaging conditions best suited for organic samples, without metallization. SEM imaging was conducted for investigation of fibrillation process as well as for statistically analyzing the distribution of formed products.

### Liposome preparation

Aqueous dispersions of liposomes (LUVs) were prepared as follows: a film of lipids was prepared on the inside wall of a round bottom flask, by evaporation of a 1 mL chloroform solution containing 1 mg of laurdan and the proper amounts of lipids. Two different kinds of LUVs were prepared: the first one was constituted by POPC and GM1 1%, while in the second one cholesterol and phosphatidylserine 10% were also added. The films obtained were stored in a desiccator overnight under reduced pressure, then 1 mL of PBS buffer solution was added to obtain a lipid dispersion. The solution was vortex-mixed and then freeze-thawed six times from liquid nitrogen to 30 °C. Dispersion was then extruded (10 times) through a 100 nm polycarbonate membrane in order to obtain Large Unilamellar Vesicles (LUVs). Extrusions were carried out at 30 °C. The vesicle size was determined by Dynamic Light Scattering (DLS) measurements using a Horiba LB550 nanoparticle size analyzer.

### Spectroscopic measurements

The binding of Aβ to LUVs was monitored through fluorescence spectroscopy. Steady-state fluorescence measurements were performed with the ISS PC1 spectrofluorimeter (ISS, Inc., Champaign, IL, USA). The binding of Aβ (5 μM) to LUVs was monitored through laurdan fluorescence incorporated in the liposomes. The probe emission spectra (λex = 360 nm, λem = 400–550 nm) were collected as a function of the [lipid]/[Aβ] ratio, indicated as [Liposome]/[Protein] ratio. For each spectra the laurdan general polarization value was calculated according to the equation (1):

1 $${GP}=\frac{{I}_{440}-{I}_{490}}{{I}_{440}+{I}_{490}}$$

Where the letter “I” is the laurdan emission fluorescence intensity at 440 or 490 nm.

### Data analysis

For electrophysiological analysis, results are reported as mean ± SEM. Statistical significance was evaluated by Student’s *t *test or one-way ANOVA followed by Turkey or Bonferroni test between 50–60 min following delivery of theta burst stimulation. Statistical significance was set at *P* < 0.05, and “*n*” was the number of brain slices used. For SEM analysis, the statistical analysis was performed using ImageJ software, with manual validation [26]. For the spectroscopic measurements, the experimental data were analysed by nonlinear regression through a hyperbolic binding isotherm in order to estimate the value of the affinity constant, [L_1/2_] as previously described [27, 28]. At this regard we used the Kaleidagraph program, version 5.0 (Synergy Software).

## Results

### Aβ fibrils affect hippocampal synaptic plasticity in ex vivo model

The effect of Aβ_1-42_ oligomers on synaptic plasticity function is largely investigated in both ex vivo and in vivo models [12–17], however poor literature describes the functional impact of Aβ fibrils on synaptic activities [8, 19], particularly in the context of their coexistence with other aggregated forms. To address this gap, we generated Aβ_1-42_ fibrils by incubating the peptide under aggregating conditions and collected samples at defined time points during fibrillization. Importantly, no purification steps were performed to isolate specific fibril species. As a result, each preparation was enriched in the dominant fibril population of each time point but retained a minor fraction of other assemblies [29]. This approach preserved the natural heterogeneity of Aβ aggregates and better mimicked the complex environment observed in pathological conditions [18, 30–34]. These fibril preparations were then applied, at the same final dilution of Aβ_1-42_ oligomers, to ex vivo brain slices to assess their effects on hippocampal synaptic plasticity [13, 14].

**Fig. 1:**
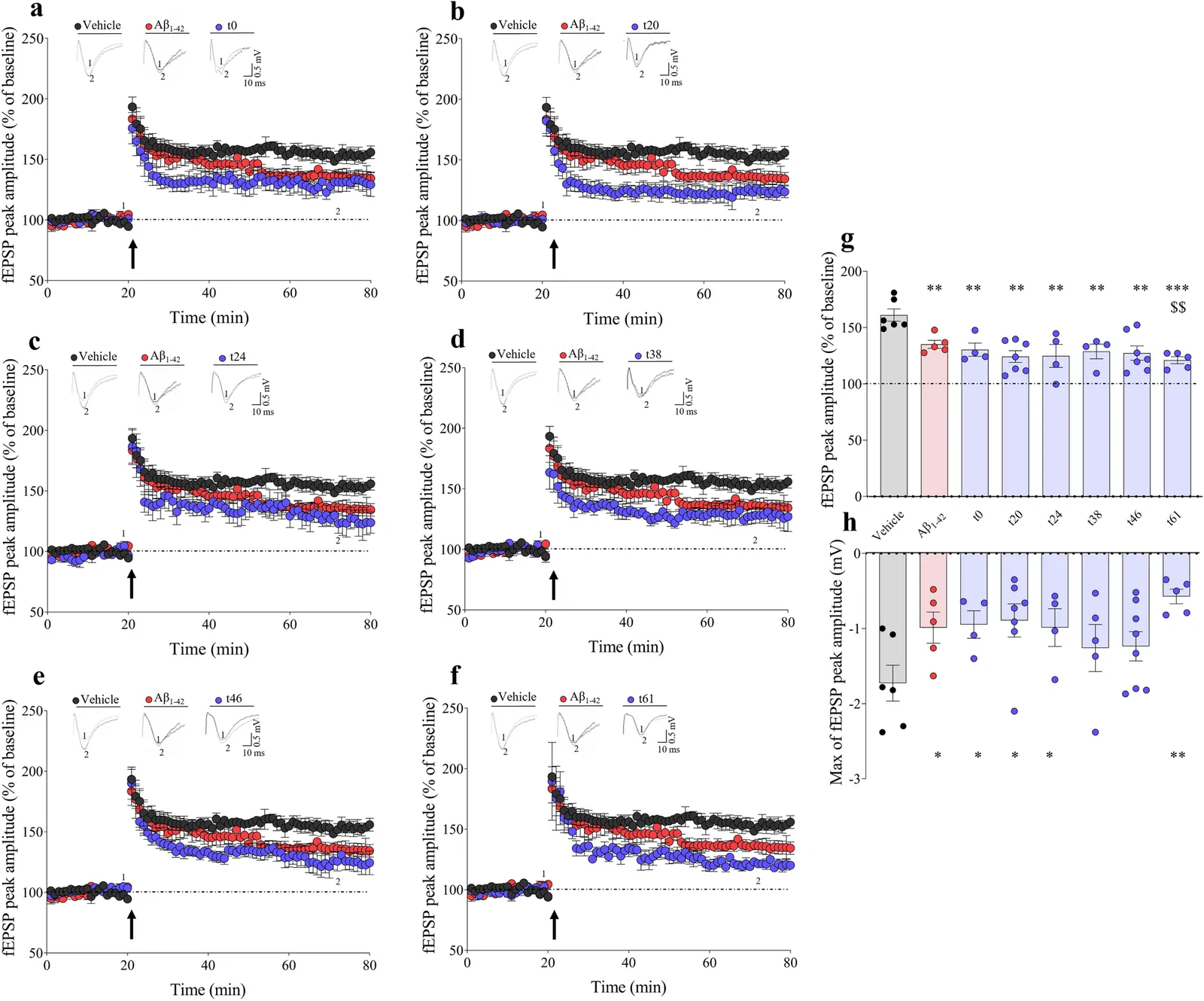
Impact of Aβ fibrils on the synaptic plasticity using an ex vivo model. Data are presented as mean value ± SEM. *P < 0.05, **P < 0.01 and ***P < 0.001 vs vehicle; ^$$^*P* < 0.01 vs Aβ_1-42_ oligomers. **a–f** Above, representative traces before (1) and after (2) the theta brust stimulation, for brain slices in the respective experimental groups: Vehicle, Aβ_1-42_ oligomers (500 nM), and the corresponding time of fibrillization (t0, t20, t24, t38, t46, t61). Below, a summary graph of the average time course of LTP. Vehicle: *n* = 6, t0 and t24: *n* = 4, t20 and t46: *n* = 7; Aβ_1-42_, t38, t61: *n* = 5. **g** Histograms illustrate the magnitude of LTP (% of baseline) in each experimental condition. Dots represent the mean of last 10 min of LTP recorded for each brain slice. **h** Histograms representing the maximal fEPSP measured after the incubation time of ex vivo hippocampal slices and before the start of baseline and LTP recording. Each dot represents the maximal fEPSP measured in a brain slice.

In our experimental conditions, Aβ_1-42_ oligomers affect synaptic plasticity and significantly reduce the magnitude of long-term potentiation (LTP) compared to the vehicle control (vehicle 161.10 ± 5.49, *n* = 6 vs Aβ_1-42_ 135.13 ± 3.52, *n* = 5, *P* < 0.01 Fig. 1a–g) [13, 14, 35]. Similarly, in the slices treated with fibrils obtained at intermediate aggregation time points (t20–t46), we observed a reduction of the LTP against the vehicle, similarly to the Aβ_1-42_ oligomers effect (t20 124.26 ± 5.25, *n* = 7, t24 124.87 ± 10.19, *n* = 4, t38 128.78 ± 6.52, *n* = 4, t46 127.35 ± 5.88, *n* = 7 vs vehicle 161.10 ± 5.49, *n* = 6, *P* < 0.01; t0–t46 vs Aβ_1-42_ 135.13 ± 3.52, *n* = 5, *P* > 0.05; Fig. 1b–e, g), suggesting equivalent synaptic-toxicity between these fibrils and Aβ_1-42_ oligomers. Conversely, in the slices treated with Aβ fibrils collected at a later aggregation stage (t61) the amplitude of potentiation was significantly reduced compared to Aβ_1-42_ oligomers treatment (t61 121.02 ± 3.17, *n* = 5 vs Aβ_1-42_ 135.13 ± 3.52, *n* = 5, *P* < 0.001, Fig. 1f, g), suggesting increased synaptic damage compared to oligomers of the same Aβ_1-42_ protein, which can be mediated by high concentrations as well as increased average fibril length and branching complexity of fibrils, as observed by SEM analysis.

To further examine synaptic integrity, we analysed baseline field excitatory postsynaptic potential (fEPSP) amplitudes before LTP induction. For the Aβ_1-42_ oligomers, we observed a significant reduction of the max amplitude recorded (Aβ_1-42_ -0.99 ± 0.21, *n* = 6 vs vehicle -1.73 ± 0.24, *n* = 5, *P* < 0.05; Fig. 1h) that was similarly observed in slices treated with Aβ fibrils t0–t46 (Fig. 1h). Consistent with the LTP findings, slices exposed to t61 fibrils exhibited a further reduction in baseline fEPSP amplitude, reinforcing the evidence of advanced synaptic damage (t61 -0.57 ± 0.10, *n* = 5 vs vehicle -1.73 ± 0.24, *n* = 5, *P* < 0.01; Fig. 1h).

**Fig. 2:**
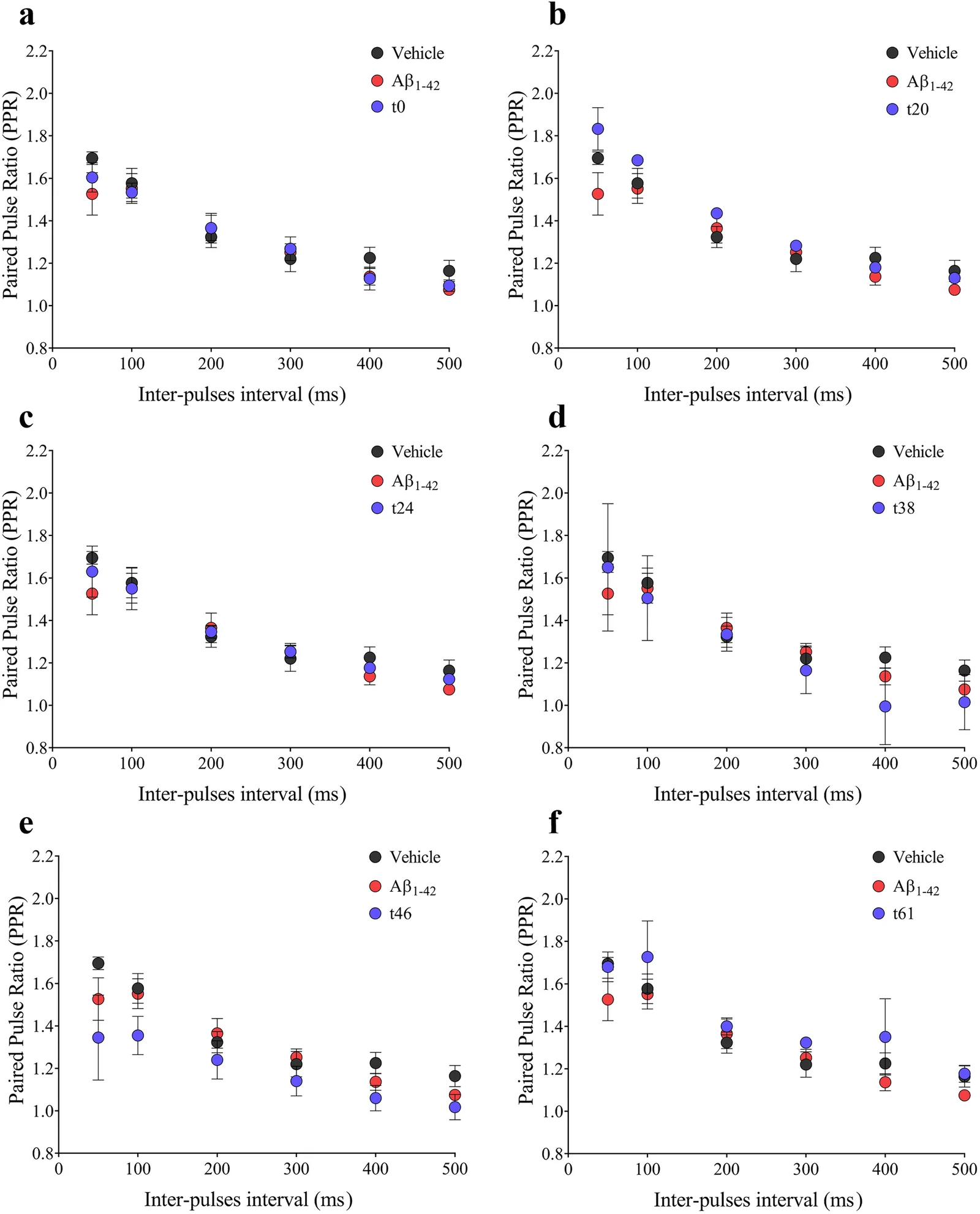
Hippocampal paired pulse ratio (PPR). PPR is induced by pairs of stimulation delivered at several interstimulus intervals (20, 50, 100, 200, 300, 500 ms) at different experimental groups: Vehicle, Aβ_1-42_ (500 nM), and the corresponding time of fibrillization (**a-f** t0, t20, t24, t38, t46, t61). Any significative change has been evaluated. Vehicle: *n* = 6, t0 and t24: *n* = 4, t20 and t46: *n* = 7; Aβ_1-42_, t38, t61: *n* = 5.

Basal neurotransmitter release was assessed using paired-pulse ratio (PPR) analysis, and no significant changes in PPR were detected across any treatment groups compared to vehicle, indicating that presynaptic neurotransmitter release is not significantly altered (Fig. 2a–f).

### Morphological assessment

**Fig. 3:**
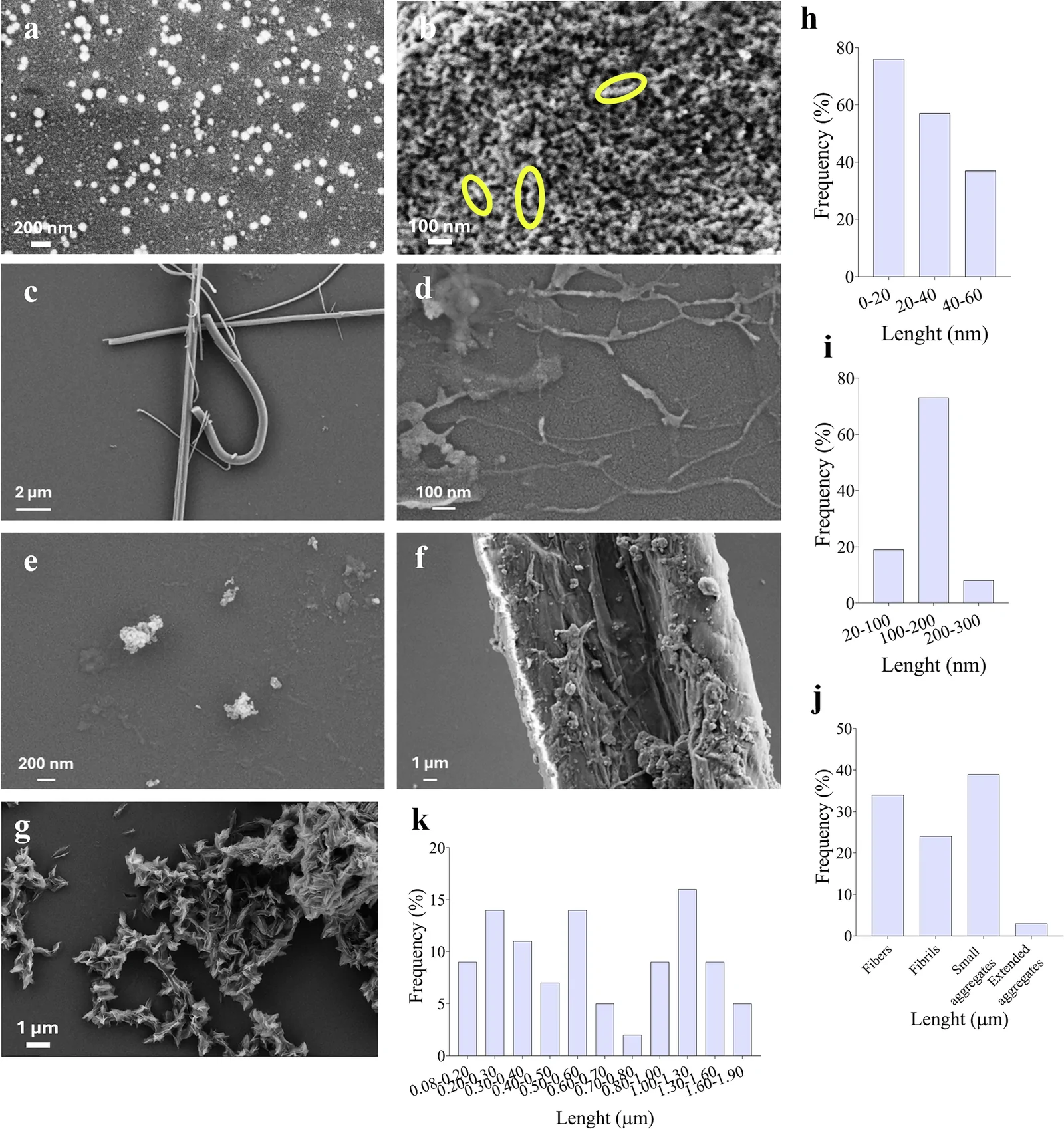
Morphological assessment of Aβ fibrils. SEM images of Aβ_1-42_ after **a** 0 h, **b** 20 h and 61 h of incubation, which reveal the presence of four distinct morphological species: **c** fibers, **d** fibrils (right), flat aggregates with extended surfaces (left), and **e** small aggregates. **f** Detailed view of a large fiber. **g** SEM images of Aβ_1-42_ after 61 h of incubation, presence of aggregates with surfaces characterized by beta-sheet formations. **h-k** Histograms show the percentage of fibrils within each length interval after **h** 0 h and **i** 20 h of incubation, where elongated oligomeric structures are highlighted in yellow; **j** the four distinct morphological species formed after 61 h of Aβ_1-42_ incubation, and **k** of fibril length distribution, showing the percentage of fibrils within each length interval (measured in μm) after 61 h of incubation.

To provide a more complete view of the aggregation process, earlier time-points were also analysed. At the initial stage (t0), only small, spherical oligomeric species were detected. These oligomers were relatively homogeneous, with radii measured in nanometers (25.50 ± 15.60, Fig. 3a, h), reflecting the earliest species formed during Aβ_1-42_ aggregation. At the intermediate stage (t20), aggregates had become progressively more heterogenous, including both spherical oligomers and elongated species, which indicates that fibril formation was beginning to occur (140.40 ± 48.50, Fig. 3b, i). Therefore, the transitional aggregates were larger than the t0 aggregates but smaller than the fibrils seen at t61; thus, the transitional aggregates represent an intermediate state between the t0 and t61 stages in the aggregate formation process, according also to the functional results of synaptic plasticity.

Consequently, based on the amplified synaptic impairment observed in ex vivo experiments at the 61 h (t61) aggregation stage, this time point was selected for detailed morphological investigation by SEM analysis, to better characterize the fibrillar structures associated with the synaptic defect described.

This analysis provided in-depth insights into the fibrillation outcomes, enabling, through statistical evaluation, to quantify different species present. Specifically, SEM images acquired after 61 h of Aβ_1-42_ incubation revealed four distinct morphological species: fibers, fibrils, small aggregates, and larger aggregates with extended surfaces (Fig. 3l).

The fibers observed at t61 are well-defined structures, with lengths ranging from 2 to 320 μm (Fig. 3c, l, m). The average fiber length is 34.4 μm, with a standard deviation of 67.3 μm, and their thickness averages 0.5 ± 1.55 μm. The fibrils appear as elongated, highly ordered structures, but with much shorter lengths compared to fibers (Fig. 3d), often appear intertwined, with smaller branching structures. As a result, the lengths of the fibrils are highly variable, ranging from 0.08 to 2 μm, with an average length of 0.7 μm ± 0.5 μm and a thickness ranging from a few nanometers to 20 nm (Fig. 3l, m).

Furthermore, the left side reveals the presence of extensive non-fibrillar aggregates (Fig. 3d). These aggregates generally have flat surfaces, though some exhibit more distinctive structures with surfaces characterized by beta-sheet formations (Fig. 3g).

Other species present are the small aggregates, which exhibit a wide range of morphologies. Some of these aggregates appear more spherical and well-defined, while others are irregularly shaped. Their size varies considerably, with diameters ranging from 0.04 to 2.23 µm. On average, these aggregates measure 0.41 µm in diameter, with a standard deviation of 0.38 µm (Fig. 3e, m).

Finally, we provide a detailed view of a large fiber, revealing its complex internal structure composed of multiple smaller fibers and fibrils. Additionally, numerous small aggregates are present both on the fiber’s outer surface, giving it a rough texture, and within its structure (Fig. 3f).

The frequency of each of the four observed species was calculated from a representative sample. Fibers represent 34%, and fibrils account for 24%, both being organized structures formed as a result of the fibrillation process. Small aggregates remain prevalent, comprising 39%, despite their reduced size. In contrast, the more extensive aggregates constitute only 3% (Fig. 3m).

### Impact of Aβ fibrils binding on liposome membrane fluidity

To mimic the cell membrane, it has been carried out a membrane model made of liposome, structurally similar to biological membranes [36], and to assess the impact of Aβ fibrils binding on liposome membrane fluidity it has been evaluated the Generalized Polarization (GP) of laurdan, a widely used parameter to assess the physical state and fluidity of lipid membranes, particularly in liposomes and biological membranes [37].

GP values reflect changes in membrane fluidity and polarity affected by the presence of proteins or peptides that alter the lipid environment and dynamics due to their binding, which can alter the lipid organization. Typically, higher GP value is usually associated with reduced membrane fluidity, low polarity, or increased cholesterol content, whereas lower GP values reflect enhanced fluidity and membrane disorder, making laurdan particularly useful for detecting transitions between liquid-ordered and disordered membrane phases [37, 38]. We used liposomes enriched with GM1, a lipid commonly found in neuronal membranes, in the presence and absence of cholesterol and phosphatidylcholine. After preparation, liposomes have been characterized by DLS and in buffer the vesicle hydrodynamic diameter was approximately 110 nm (data not shown).

**Fig. 4:**
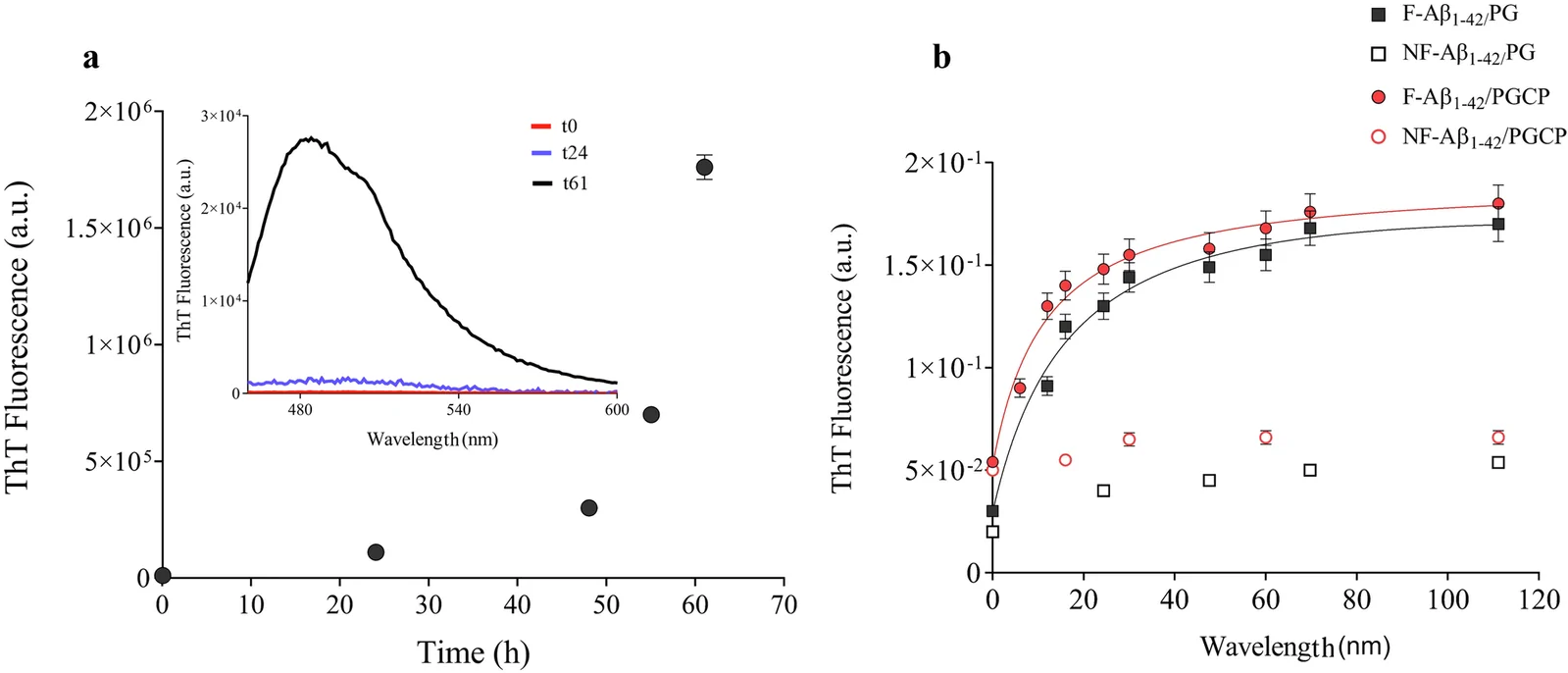
Aβ fibrils affect membrane fluidity of liposome. **a** Fluorescence of ThT in the presence of Aβ_1-42_ incubated at 37 °C for different time. The inset shows the ThT spectra in the presence of Aβ_1-42_ that was freshly prepared (red) or incubated for 24 h (black) and 61 h (blue) at 37 °C. **b** Filled and open symbols indicate the effect of Aβ_1-42_ fibrils (F-Aβ_1-42_) on liposome membrane fluidity compared to non-fibrillated Aβ_1-42_ (NF-Aβ_1-42_). The experiments were carried out on LUVs with two different compositions: POPC and GM1 (black) (PG), and POPC–GM1–cholesterol–phosphatidylcholine (red) (PGCP). Data are shown as a function of the ratio between liposomes and Aβ_1-42_ fibrils (5 μM) concentrations. The solid lines correspond to the best fit obtained described in “Materials and methods” section.

To assess membrane binding, we first incubated the Aβ_1-42_ peptide at 37 °C and monitored fibril formation over time by measuring changes in ThT fluorescence intensity (see “Materials and methods”). A substantial increase in the probe’s fluorescence intensity, indicative of fibril formation, was observed only after 48 h of incubation (Fig. 4a). As illustrated in Fig. 4b, the membrane interaction properties of Aβ_1-42_ differ according to its quaternary structure.

In our conditions, incubation with Aβ₁₋₄₂ oligomers didn’t significantly affect laurdan GP value, suggesting minimal or no interaction with the membrane under these conditions (Fig. 4b) for both types of liposome compositions tested. In contrast, treatment with Aβ_1-42_ fibrils (i.e., Aβ incubated for 61 h at 37 °C) resulted in a clear increase GP value in a dose-dependent manner, indicating a reduction in membrane fluidity (Fig. 4b), confirming the evidence that fibrils interact with GM1 rich domain within membranes, potentially contributing to membrane rigidity and neuronal dysfunction [39]. Obviously, the reduction in fluidity varies depending on the two types of membranes, since the liposomes containing cholesterol and phosphatidylcholine have a higher GP value, indicating lower initial fluidity. These data allowed us to calculate, by nonlinear regression analysis, the values of the affinity constant, [L_1/2_] obtained for the two different liposomes compositions. The parameters obtained by fitting the curves results to be [L_1/2_] =(50 ± 12) μM and [L_1/2_] =(32 ± 10) μM in the case of liposomes with or without cholesterol respectively. These two values do not differ significantly (*P* ≈ 0.25) indicating that the propensity of Aβ_1-42_ fibrils to bind membranes is not affected by liposome composition, at least for these two conditions.

## Discussion

There is increasing evidence supporting the synaptic toxic properties of the various aggregation states of Aβ, including monomers, oligomers, protofibrils, and mature fibrils, in the context of Alzheimer’s disease [9, 12]. The exact mechanisms by which Aβ fibrils may induce synaptic dysfunction are still not completely characterized, and Aβ oligomers have been assumed to be the harmful species [40–42]. The term oligomer refers to a heterogeneous group of Aβ assemblies that vary widely in size and structure, leading to ongoing debate about which species are most neurotoxic. Among them, small Aβ oligomers may be some of the most dangerous assemblies because they can rapidly diffuse due to their sizes and are likely to have highly reactive hydrophobic surfaces [18, 43]. These features cause membrane damage and make them easier to enter cells, promoting the spread of toxic aggregates [44].

In line with this, larger prefibrillar Aβ_1-42_ oligomers are considered the most synaptotoxic species, because they integrate into the lipid bilayer, forming abnormal ion-permeable pores that allow uncontrolled calcium influx, perturbing cellular homeostasis, and leading to neuronal toxicity [45–47].

Nevertheless, the interest in amyloid fibers and fibrils, as key disease mediators, has seen a huge increase in recent years, also given the complex environment observed in pathological conditions [18, 30, 31, 48]. In this context, our approach which does not include purification into fibrils species, preserves such complexity that more closely mimics the pathological environment, where different Aβ assemblies coexist and interact. Specifically, our results reveal that mature fibrils and fibers also exert significant synaptic-toxic effects in an ex vivo hippocampal model, as we observed that acute exposure to Aβ fibrils and fibers impair long-term potentiation (LTP), a key mechanism of synaptic plasticity and memory formation [49]. Specifically, under our experimental conditions, a strong reduction in synaptic plasticity was observed in association with increased fibril structural complexity at t61. Indeed, at this time point, the SEM analysis fibrils displayed significantly increased average length and branching density, indicative of an advanced maturation state, together with enhanced β-sheet content. This prompts that functional deficits observed are linked to fibril maturation and structural complexity rather than peptide concentration alone, as demonstrated by a similar synaptic plasticity defect mediated by protein oligomeric form and fibril forms until t61. The morphological analysis further revealed a mixture of Aβ species, including mature fibrils and fibers, protofibrils, and small aggregates. This heterogeneity also suggests that synaptic impairment likely reflects contributions from all aggregate forms, rather than being exclusively caused by mature fibrils, while the enhanced effects at later stages of aggregation indicate that structural maturation of fibrils plays an important role, pointing to an aggregation state-dependent neurotoxicity. Beyond the co-presence of several forms of Aβ aggregates, the possibility of the co-interaction between these different structures might further contribute to the synaptic impairment observed in our model. For instance, fibrillar structures, in their network, can sequester or locally concentrate the oligomeric form at the membrane level, potentially enhancing the synaptic toxicity [32–34]. On the other hand, conversely, fibrils might transiently buffer the toxicity by incorporating them in higher assemblies, while environmental factors, such as lipid composition and pH, already affected, can induce their release in other neurotoxic forms[32–34]. Such synergistic or antagonistic interactions between aggregate states could generate emergent toxic effects that are not predictable from individual species alone, offering a more dynamic view of how heterogeneous Aβ assemblies collectively drive synaptic dysfunction.

Consequently, the complex profile of different aggregate state species suggests that all mixture forms may contribute to the observed synaptic toxicity, reflecting the complexity of the pathological brain environment characterized by copresence of different Aβ states [48, 50, 51], with emerging methods quantifying diverse structural forms and modeling approaches that explicitly consider transitions between monomeric, oligomeric, and fibrillar states [52, 53]. In this regard, protein aggregation is a dynamic process characterized by the formation of several transient intermediate structures, including small aggregates, fibrils, and fibers [48, 50]. In particular, the fiber can represent a reservoir sequestering the oligomeric forms and then reducing the toxicity of the oligomeric presence. In contrast, increasing the length of amyloid fibrils affects cell membrane interaction [39] and function [54]. On the other hand, the pathological process occurring in AD is a very long process characterized by several factors that can influence the dynamics of plaque formation. Notably, change in brain environment, such as the acidic pH shift during neuroinflammatory process, which represents a common feature of neurodegenerative disease, facilitates the protein misfolding and aggregation [55–57].

However, a direct correlation between cognitive decline and Aβ aggregation state is unknown. Observation of the amyloid plaque rich in Aβ fibrils in AD brain supported the hypothesis of a central role of protein aggregation in neurodegeneration. Different studies conducted using several approaches, including in vitro and in vivo models, demonstrated that Aβ fibrils induced alterations similar to AD brain [19, 34, 58]. On the other hand, a lot of investigation has been conducted using Aβ oligomers showing neurotoxicity in line with the oligomer hypothesis [9, 11, 16, 20]. All this evidence confirms the complexity of AD conditions in which there is a co-presence of several Aβ structures, each responsible of a damage level [10, 13, 14, 19], suggesting again the importance to include each of them in this type of studies.

Although the impact of Aβ fibrils on neuronal function has been less extensively characterized, several studies suggest that fibrils directly affect membrane properties [4, 59]. In particular, high molecular weight (HMW) fibrils induce greater reduction in membrane fluidity than low molecular weight (LMW) counterparts, likely affecting membrane protein function, neurotransmission, and receptor dynamics [60]. Our results are consistent with these observations, confirming that fibrils impact on the membrane fluidity according to literature [48, 58, 61]. Additionally, the influence of membrane composition was established as an important means to configure Aβ_1-42_ toxicity and cholesterol, which can modify membrane rigidity, both exhibited effects on the disruption of membrane integrity and levels of cellular stress [59]. The lipid-mediated specific effects may modulate Aβ toxicity by modifying the structure of fibrils, binding affinity, and subsequent downstream signaling [59]. With respect to membrane composition, fibrils were also related to the activation of cellular stress programs such as the UPR, calcium dysregulation, and oxidative damage, all of which are important drivers of AD pathology [62, 63]. Our findings confirm the interaction between liposome, containing GM1, and the fibrils having an impact on membrane fluidity, which it can be reflected also at functional level as demonstrated by LTP impairment.

From a pharmacological standpoint, all these findings have notable implications, indeed new drugs, such as Lecanemab, target soluble protofibrils, underscoring the therapeutic relevance of early aggregation intermediates [23]. However, our findings indicate that mature fibrils, even though they seem less mobile and are often thought to be inert, play an active role in causing synaptic dysfunction and damaging membranes. Moreover, their persistence in the brain and interactions with neuronal lipids make them as viable pharmacological targets, supporting the idea that strategies aimed at preserving membrane integrity, modulating lipid composition, or disrupting fibril–lipid interactions could offer complementary therapeutic avenues alongside current oligomer-targeted strategies.

In conclusion, this study supports the concept that Aβ fibrils are not passive end-products of aggregation but active contributors to synaptic and cellular damage in AD. As a result, their toxicity is altered by aggregation state, and lipid microenvironment, complicating the pathological cascade even further, and suggesting the notion that oligomers as the primary progenitor of the pathological process needs to be reevaluated. Further, understanding regarding how different Aβ assemblies interact with neuronal membranes is critical if we are to develop feature therapeutics and seek out new pharmacological targets.
